# Understanding the Landscape: The Emergence of Artificial Intelligence (AI), ChatGPT, and Google Bard in Gastroenterology

**DOI:** 10.7759/cureus.51848

**Published:** 2024-01-08

**Authors:** Rajmohan Rammohan, Melvin V Joy, Sai Greeshma Magam, Dilman Natt, Sai Reshma Magam, Leeza Pannikodu, Jiten Desai, Olawale Akande, Susan Bunting, Robert M Yost, Paul Mustacchia

**Affiliations:** 1 Gastroenterology, Nassau University Medical Center, East Meadow, USA; 2 Internal Medicine, Nassau University Medical Center, East Meadow, USA; 3 Gastroenterology and Hepatology, Nassau University Medical Center, East Meadow, USA

**Keywords:** liver cirrhosis, openai, pancreatitis, acute pancreatitis, gastroenterology, google bard, chatgpt

## Abstract

Introduction

Artificial intelligence (AI) integration in healthcare, specifically in gastroenterology, has opened new avenues for enhanced patient care and medical decision-making. This study aims to assess the reliability and accuracy of two prominent AI tools, ChatGPT 4.0 and Google Bard, in answering gastroenterology-related queries, thereby evaluating their potential utility in medical settings.

Methods

The study employed a structured approach where typical gastroenterology questions were input into ChatGPT 4.0 and Google Bard. Independent reviewers evaluated responses using a Likert scale and cross-referenced them with guidelines from authoritative gastroenterology bodies. Statistical analysis, including the Mann-Whitney U test, was conducted to assess the significance of differences in ratings.

Results

ChatGPT 4.0 demonstrated higher reliability and accuracy in its responses than Google Bard, as indicated by higher mean ratings and statistically significant p-values in hypothesis testing. However, limitations in the data structure, such as the inability to conduct detailed correlation analysis, were noted.

Conclusion

The study concludes that ChatGPT 4.0 outperforms Google Bard in providing reliable and accurate responses to gastroenterology-related queries. This finding underscores the potential of AI tools like ChatGPT in enhancing healthcare delivery. However, the study also highlights the need for a broader and more diverse assessment of AI capabilities in healthcare to leverage their potential in clinical practice fully.

## Introduction

Artificial intelligence (AI) has significantly transformed healthcare, including gastroenterology. Its application extends beyond traditional business and societal roles, enhancing patient care, medical decision-making, and pharmaceutical development. Numerous studies back AI's potential to outperform humans in various healthcare tasks [1].

In gastroenterology, AI plays a crucial role in diagnosing and treating diseases. It aids clinicians by identifying and classifying polyps and tailoring treatment plans based on patient data, thereby improving outcomes and minimizing side effects. AI also contributes significantly to cancer detection, especially in pancreatic and esophageal cases [2].

AI's ability to diagnose and treat interconnected, often asymptomatic, digestive diseases marks a breakthrough in medical technology. Enhanced diagnostic sensitivity and accuracy in liver, gastric, pancreas, and colon diseases demonstrate AI's impact. Innovations like ChatGPT and studies like Hirasawa et al., which showcased AI's proficiency in gastric cancer diagnosis, underscore AI's growing importance and potential in medical research and real-time clinical applications [3].

This article was previously presented as a meeting abstract at the 2023 American College of Gastroenterology (ACG) Conference Meeting at Vancouver on October 22, 2023.

## Materials and methods

Data extraction

ChatGPT and Google Bard were tested in this study using a series of typical gastroenterology questions a patient might ask. These questions were input into each AI tool and prefaced with the phrase, "What's the suitable response for the following?". Each question was presented in a new chat session to maintain the experiment's integrity and prevent any influence on memory retention. The responses generated by ChatGPT and Google Bard were then meticulously analyzed by two independent reviewers who were not aware of which AI tool produced which response. These reviewers employed the Likert scale for evaluation, where a score of 1 indicated a "poor" response and a score of 10 represented an "excellent" response. This scoring system provided a standardized method to assess the quality of the AI-generated answers.

The accuracy of the responses was determined by cross-referencing them with guidelines from authoritative bodies in the field, such as the ACG, the American Gastroenterological Association (AGA), and the American Association for the Study of Liver Diseases (AASLD). This comparison was critical to ascertain how closely the AI responses aligned with current medical standards and practices. Additionally, the reliability of the AI tools was evaluated based on several criteria: the user interface of the AI platforms, the ease of accessing these tools, and the promptness of their responses. This comprehensive assessment aimed to provide a thorough understanding of both the accuracy and reliability of ChatGPT and Google Bard in answering medical queries related to gastroenterology.

Analysis

Descriptive Statistics

The initial phase of the analysis involved descriptive statistics to summarize the central tendencies and variabilities of the ratings for both AI systems. Key metrics calculated included the mean, which provided an average rating for each system; the median, highlighting the central tendency of the dataset; the standard deviation, offering insights into the variability and spread of the ratings; and the range (minimum and maximum values), indicating the extent of ratings received. These statistics were instrumental in providing an initial overview of the data, enabling a clear understanding of the general trends and differences in ratings between ChatGPT 4.0 and Google Bard.

Hypothesis Testing

Hypothesis testing was conducted to assess the significance of the observed differences in ratings between the two AI systems. The Mann-Whitney U test, a non-parametric test ideal for comparing two independent samples, was chosen due to its robustness against non-normal data distributions and unequal variances. This test was applied separately to the reliability and accuracy of datasets. The resultant p-values from these tests provided a statistical basis to determine whether the differences in ratings were significant, with values less than the conventional alpha level of 0.05 indicating statistically significant differences.

Combining both descriptive and inferential statistics, this comprehensive analytical approach provided valuable insights into the comparative performance of ChatGPT 4.0 and Google Bard regarding reliability and accuracy as perceived by the respondents. While the descriptive statistics offered a clear overview of the data, the hypothesis testing added a layer of statistical rigor, confirming the significance of the observed differences.

Data processing software

For the statistical analysis in this study, data processing and calculations were conducted using Microsoft Excel (Version 16.73, Microsoft Corporation, Redmond, Washington, United States) and IBM SPSS Statistics for Windows, Version 29.0 (Released 2022; IBM Corp., Armonk, New York, United States). The analytical approach involved performing repeated unpaired two-sample t-tests to compare the means between the two groups under examination. The significance of the results was determined based on a two-tailed p-value approach, with a threshold set at less than 0.05 to denote statistical significance. This methodology ensured a robust and reliable data analysis, providing a solid foundation for the study's conclusions.

Ethics approval

This research evaluated the public contributions and perceptions regarding the quality of ChatGPT and Google Bard, thus deeming the approval of an ethics committee non-essential.

## Results

A comprehensive comparison of ChatGPT 4.0 and Google Bard, segmented into two primary dimensions (reliability and accuracy), is shown in Table 1 and Table 2. These dimensions are crucial in understanding and evaluating the performance of these advanced AI systems.

**Table 1:** Reliability questionnaire AI: artificial intelligence

	Ease of use
1	The interface of the AI is intuitive and user-friendly.
2	I could easily navigate and utilize the features of the AI.
3	Learning to use the AI was straightforward and did not require extensive training.
4	The AI provided clear and understandable responses.
5	I could easily integrate the AI into my existing workflow.
	Performance
1	The AI consistently provided accurate information.
2	The responses from the AI were relevant and useful for medical management.
3	The AI functioned without significant errors or issues.
4	I trust the information and guidance provided by the AI.
5	The AI maintained a consistent performance level during different tasks.
	Response time
1	The AI provided responses in a timely manner.
2	The speed of the AI's responses did not hinder my work.
3	I am satisfied with the AI's efficiency in handling queries.
4	The AI's response time was consistent across different types of tasks.
5	The response time of the AI met my expectations for a quick turnaround.
	Overall experience
1	I am satisfied with the overall experience of using the AI.
2	The AI met my needs for medical management.
3	I would recommend the AI to other professionals in my field.
4	The AI added value to my work process.
5	I prefer using AI over traditional methods for medical management tasks.

**Table 2:** Accuracy questionnaire GI: gastrointestinal; *H. pylori*: *Helicobacter pylori*; GERD: gastroesophageal reflux disease

	Questions
1	Write the management for acute pancreatitis.
2	Write the management for alcoholic liver disease.
3	How do you treat irritable bowel disease?
4	What is the management of *H. pylori*?
5	How do you treat upper GI bleeding?
6	What is the initial management for lower GI bleeding?
7	How do you diagnose GERD?
8	How do you evaluate dysphagia?

Part 1: reliability assessment

Reliability, in this context, refers to the consistency and dependability of the AI systems in performing tasks or providing information.

*Statistical Overview of Reliability* 

The dataset reveals some interesting patterns in the reliability ratings of both AI systems. ChatGPT 4.0 exhibits a higher average rating mean of 6.23 and a more significant response variability standard deviation of 5.51. This suggests that while ChatGPT 4.0 is generally considered more reliable, opinions vary widely among users. In contrast, Google Bard displays a lower average reliability rating mean of 2.04 with a less variability standard deviation of 1.82, indicating a more consistent but lower perceived reliability.

Hypothesis Testing on Reliability

**Figure 1:**
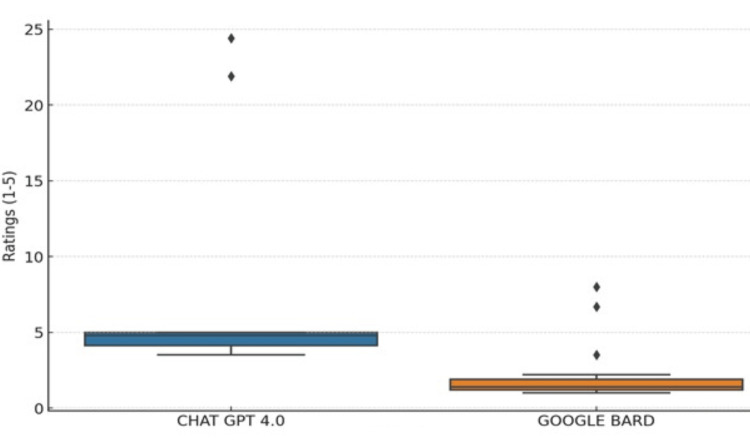
Box plot for reliability ratings

To analyze these observations further, a Mann-Whitney U test was applied. This non-parametric test compares differences between two independent groups when the dependent variable is ordinal or continuous but not normally distributed. The test revealed a statistically significant difference in the reliability ratings between the two AI systems, with a p-value of approximately <0.01. This indicates that users consistently rated ChatGPT 4.0 higher in reliability than Google Bard, as shown in Figure 1.

Part 2: accuracy assessment

Accuracy pertains to how close the AI systems are to providing information or results that are correct or truthful.

Statistical Overview of Accuracy

In accuracy, ChatGPT 4.0 again leads with a higher mean rating of 4.48 and a standard deviation of 0.47, implying that it is generally considered more accurate with less response variability. Google Bard, on the other hand, has a lower average accuracy rating mean of 2.48 and a standard deviation of 0.35, suggesting a lower overall accuracy with less fluctuation in user ratings.

Hypothesis Testing on Accuracy

**Figure 2:**
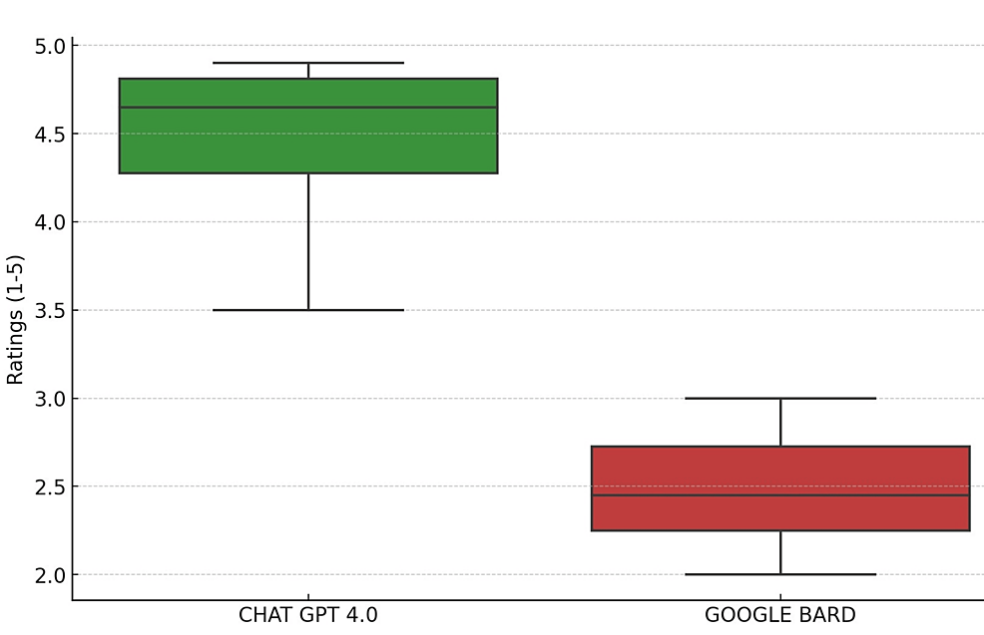
Box plot for accuracy ratings

**Figure 3:**
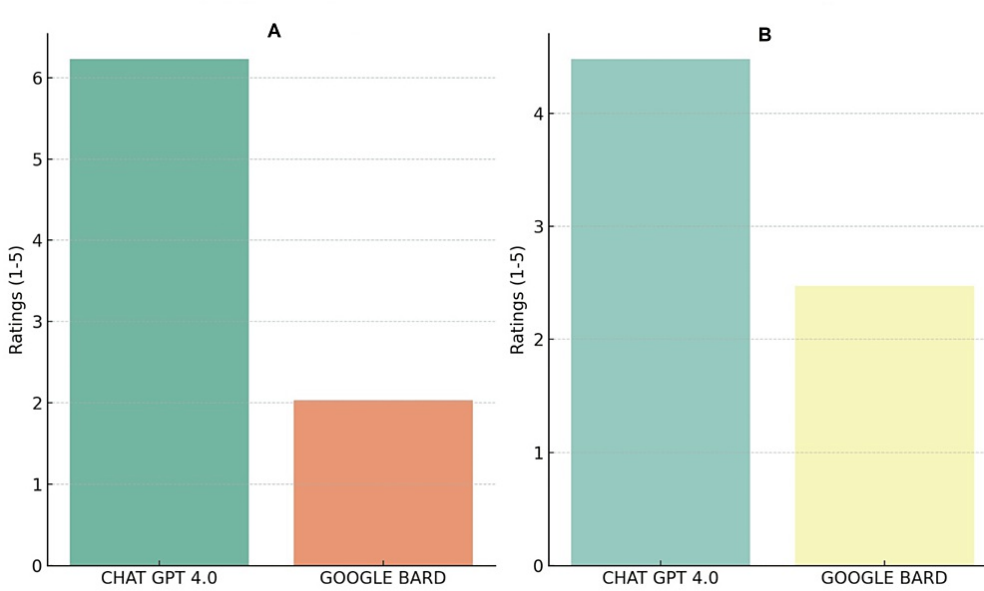
Comparative outcome between the AI models: (A) reliability outcome and (B) accuracy outcome AI: artificial intelligence

A Mann-Whitney U test was also conducted for the accuracy ratings. The results echoed the reliability analysis, showing a statistically significant difference between the two AI systems, with ChatGPT 4.0 being rated as more accurate. The p-value in this case was approximately 0.00016, reinforcing the observed trend as shown in Figure 2. Figure 3 shows the comparative outcome between the AI models.

## Discussion

Brief overview

AI has been increasingly integral in medicine for the past two decades, notably in gastroenterology and hepatology [4]. Central to AI is machine learning, which harnesses pattern recognition from existing data to enhance the analysis of new information [4]. Clinically, AI proves vital in detecting both premalignant and malignant lesions, including conditions like Barrett's esophagus, esophageal squamous cell carcinoma, and gastric cancer [4]. Its utility extends to predicting treatment outcomes, tailored to individual patient profiles and specific treatment contexts [1]. Noteworthy research includes Martin et al.'s 2020 study, where AI was used to diagnose *Helicobacter pylori* infection through histopathological imaging [5]. Similarly, a 2017 study demonstrated AI's role in diagnosing the same infection, revealing a significant decrease in diagnostic time and increased accuracy [6]. These advancements underscore AI's growing impact and potential in enhancing diagnostic precision and treatment efficacy in the medical field.

Role in diagnostics 

ChatGPT, a prominent AI tool, has gained considerable attention recently [7]. However, its reliability and accuracy, particularly when responding to open-ended medical questions, have not been thoroughly evaluated [7]. To address this gap, a 2023 study focused on assessing the accuracy and reliability of search engines like ChatGPT in the context of medical inquiries [7]. The study encompassed a range of easy, medium, and hard questions [7]. The findings indicated that the average accuracy of ChatGPT responses fell within the spectrum of nearly to completely correct [7]. These results highlight the critical need for ongoing evaluation of such accessible search engines, ensuring that the medical information they provide remains trustworthy and useful for patients [7].

Information processing and management

Integrating AI in managing patient records and health information is becoming increasingly prominent. AI's primary utility in this context lies in extracting pertinent health information efficiently. A notable study involving over 500 primary care physicians demonstrated the effectiveness of AI in processing faxed records from various clinical practices [8]. This study revealed that physicians spend about 62% of their time reviewing electronic health records (EHR). The introduction of AI-optimized record extraction significantly reduced this time, saving an average of 2.3 minutes per review, which equates to an 18% reduction in standard review time [8]. For new patient encounters, the time savings averaged 14.5 minutes. The study's findings were overwhelmingly positive, with 11 out of 12 participating physicians expressing a preference for incorporating AI software into their clinical practices, indicating its potential to streamline healthcare processes and enhance efficiency in patient care management [8].

Patient interaction and support

The advancement of information technology, especially in AI applications, is significantly enhancing patient-centered self-management in healthcare. Implementing reminders and alerts linked to these technologies has improved health outcomes. Patients are increasingly adhering to healthcare professionals' advice, thanks partly to the support and guidance offered by AI-driven tools [9]. Research indicates that applications and online portals facilitate better communication between patients and their healthcare providers, boosting engagement rates by 60% or more. These AI applications assist in effective communication and play a crucial role in educating patients about non-emergency health issues. Consequently, these technological innovations are pivotal in promoting patient engagement and adherence to treatment plans, effectively reducing the workload and burden on healthcare providers. This trend underscores the growing importance of integrating technology into healthcare strategies for better patient outcomes [2].

Research and development in gastroenterology 

Drug Discovery

The immense chemical space, with over 10^60^ molecules, offers a vast potential for drug development [10,11]. Yet, traditional drug discovery methods are often hindered by their time-consuming and costly nature [10]. AI presents a solution to these challenges, capable of rapidly identifying hit and lead compounds and streamlining drug target validation and structural optimization [11,12]. A notable example is the work of researchers at Nagoya University in Japan, who utilized AI to develop a new gastric acid inhibitor [13]. Focusing on the gastric proton pump's steric structure, they employed "Deep Quartet", an AI-driven drug discovery platform, to analyze this complex protein [14]. This led to DQ-18, a compound with a binding affinity almost 10 times higher than the prototype gastric acid inhibitor SCH28080 [14]. This breakthrough underscores the crucial synergy between human expertise and AI in drug discovery, marking a significant advancement in pharmaceutical development [14]. It promises more effective treatments for gastric acid-related conditions and inspires novel drug discovery methodologies [14].

Clinical Research

Clinical trials, essential for determining a drug's safety and efficacy in treating specific diseases, typically span six to seven years and demand significant financial resources [15]. Despite this investment, only about one in 10 tested molecules successfully passes these trials, leading to considerable industry losses [15]. Many of these failures stem from inadequate patient selection, lack of technical resources, and subpar infrastructure [16]. The vast amount of digital medical data available presents an opportunity to mitigate these challenges [16]. Implementing AI in this context can significantly reduce the failure rate in clinical trials, optimizing patient selection, enhancing technical capabilities, and improving overall trial infrastructure [16]. AI integration could revolutionize drug development processes' efficiency and success rates [15,16].

Ethical considerations

Data Privacy and Security

In the healthcare sector, AI is revolutionizing medicine and addressing major global healthcare challenges [17]. A notable example is AlphaFold, an AI algorithm that resolved the decades-old protein folding problem, significantly impacting biology and medicine [18]. Additionally, advancements like in silico trialing enable pharmaceutical companies to simulate clinical trials digitally, optimizing drug development with greater population models and reduced resource usage [17].

However, with these advancements come concerns about medical privacy, which involves safeguarding patient records and ensuring confidentiality in healthcare interactions [17]. This concept extends to protecting patients' privacy within medical facilities and maintaining modesty during medical procedures [17]. The rise of patient care management systems (PCMS) and EHR brings new privacy challenges, balancing data security with reducing redundant services and medical errors [19].

In the context of machine learning and big data, privacy protection is crucial to guard against malicious attacks aimed at extracting sensitive information, thus preventing unintentional data disclosure [20]. Techniques like federated learning and hybrid techniques and an understanding of potential privacy attacks and security challenges are pivotal in shaping future directions in this field [20].

Bias and Discrimination

The issue of data exploitation, defined as the unauthorized use of personal data, is increasingly prevalent in today's digital age [21]. Many consumer products, including smart home devices and computer software, are equipped with features that allow for data mining through AI models [21]. A large segment of the population is unaware of how extensively their devices and apps collect, process, and share data, leading to privacy violations [21]. Despite these risks, there is a rising demand for remote monitoring systems, particularly in healthcare [21]. Wearable devices that track vital health parameters like blood pressure, glucose levels, and heart rate are becoming more common [21]. As reliance on digital technology grows, so does the potential for data exploitation, posing a growing threat to user privacy [21]. This trend underscores the need for increased awareness and protective measures against the unauthorized use of personal data [21].

Limitations of the study

The study, while insightful, faces notable limitations. The reliance on a specific set of gastroenterology-related questions may not comprehensively represent the full scope of AI capabilities in diverse medical scenarios. Additionally, the data structure constrained the analysis, particularly the inability to conduct a detailed correlation analysis, which could have provided deeper insights into the nuances of AI performance. The exclusive focus on ChatGPT 4.0 and Google Bard also limits the generalizability of the findings, as it excludes other emerging AI tools that might offer different or complementary insights in gastroenterology.

## Conclusions

This comprehensive study highlights the significant role of AI in gastroenterology, emphasizing its potential to improve diagnostic accuracy and treatment efficacy. The analysis of ChatGPT 4.0 and Google Bard using gastroenterology-related queries reveals distinct differences in their performance. ChatGPT 4.0 demonstrates superior reliability and accuracy, as evidenced by higher average ratings and statistically significant differences in hypothesis testing. However, limitations in data structure precluded a detailed correlation analysis. The findings underscore the importance of AI in enhancing patient care in gastroenterology, with tools like ChatGPT showing promise in providing accurate medical information. This study affirms the growing relevance of AI in healthcare and highlights the need for continuous evaluation and improvement of these technologies to ensure their reliability and utility in clinical settings.
